# Development of a Scheme and Tools to Construct a Standard Moth Brain for Neural Network Simulations

**DOI:** 10.1155/2012/795291

**Published:** 2012-08-16

**Authors:** Hidetoshi Ikeno, Tomoki Kazawa, Shigehiro Namiki, Daisuke Miyamoto, Yohei Sato, Stephan Shuichi Haupt, Ikuko Nishikawa, Ryohei Kanzaki

**Affiliations:** ^1^School of Human Science and Environment, University of Hyogo, 1-1-12 Shinzaike-Honcho, Himeji, Hyogo 670-0092, Japan; ^2^Research Center for Advanced Science and Technology, The University of Tokyo, Tokyo 153-8904, Japan; ^3^College of Information Science and Engineering, Ritsumeikan University, Kusatsu, Shiga 525-8577, Japan

## Abstract

Understanding the neural mechanisms for sensing environmental information and controlling behavior in natural environments is a principal aim in neuroscience. One approach towards this goal is rebuilding neural systems by simulation. Despite their relatively simple brains compared with those of mammals, insects are capable of processing various sensory signals and generating adaptive behavior. Nevertheless, our global understanding at network system level is limited by experimental constraints. Simulations are very effective for investigating neural mechanisms when integrating both experimental data and hypotheses. However, it is still very difficult to construct a computational model at the whole brain level owing to the enormous number and complexity of the neurons. We focus on a unique behavior of the silkmoth to investigate neural mechanisms of sensory processing and behavioral control. Standard brains are used to consolidate experimental results and generate new insights through integration. In this study, we constructed a silkmoth standard brain and brain image, in which we registered segmented neuropil regions and neurons. Our original software tools for segmentation of neurons from confocal images, KNEWRiTE, and the registration module for segmented data, NeuroRegister, are shown to be very effective in neuronal registration for computational neuroscience studies.

## 1. Introduction

Insect brains are important model systems for analyzing neural function. This is due to their comparatively simple structure incorporating important brain functions such as sensory information processing, learning, and behavioral control mechanisms [1–3]. Analysis based on the morphologies of neurons and neuropils has greatly promoted the understanding of neural function. In particular, the existence of numerous identified neurons has consolidated the application of insect brains as model neural networks in the field of neuroethology [4, 5]. The detailed morphology of neurons can be captured more readily using recent fluorescence techniques and various genetic technologies in insects [6–9]. These methodological advances have resulted in new insights into brain mechanisms through the use of small and tractable insect brains.

A well-known simple insect behavior is the unique orientation to pheromone stimuli displayed by the male silkmoth, *Bombyx mori*. This programmed behavior triggered by sensing pheromone consists of surge, zigzag, and looping locomotor components [10]. Sensory signal pathways for pheromone have already been identified and characterized by intra- and extracellular experiments. However, these results are still insufficient to obtain a global understanding from sensory processing to behavioral control mainly owing to experimental limitations.

For example, the lateral accessory lobe (LAL) and the ventral protocerebrum (VPC) are considered to be key regions for generating command signals to control moth behavior [10]. In these regions, unique flip-flop neural responses thought to be related to the zigzag behavior were recorded, and their neural substrates were analyzed in detail morphologically [11]. By modeling a neural network based on experimental data and simulating it under natural environmental conditions in real time, precise hypotheses can be tested to gain crucial insights into the neural mechanisms generating behavior. Using a supercomputer, we are developing a simulation model for the whole neural pathway based on the real neural structure, properties, and connections. Even using the fastest computer currently available, an insect brain of ca. 100,000–1,000,000 neurons simulated using detailed neuronal properties will test available computational power to the limit.

In brain science, standard brain maps are used to integrate and compare morphological data taken from different subjects at different times. Standard brains have already been developed for various insect species, such as *Drosophila* [12–14], the honeybee [15, 16], two moth species [17, 18], and the locust [19, 20]. These standard brains have been employed for various morphological analyses of neurons and brain regions. For example, possible synaptic connections between identified neurons have been analyzed using the honeybee standard brain [21], while morphological development of the optic lobes has been studied in a moth brain [22].

Brain functions are generally thought to be generated by the dynamics of neuronal responses. These dynamics are controlled by various factors, such as ion channels, intracellular signaling, and neuronal morphology. In order to analyze the dynamical properties of neurons and their networks in the silkmoth, we have been integrating our experimental data into a database [23], which contains more than 1,200 single-neuron records of morphological and physiological experimental data. To take advantage of the registered information to build a computational model for investigating neural mechanisms, we developed a method and tools for constructing and utilizing the standard brain.

The outline of the silkmoth standard brain was constructed by averaging brain images followed by binarization. Brain images, segmented regions, and neurons can be registered in it by a nonrigid transform. Our original tools for segmentation of neurons from confocal images, KNEWRiTE, and a registration module for Fiji and ImageJ for neuron morphological data, NeuroRegister, were effective in conducting this registration process. Neural simulations linked to the standard brain are started by registering neurons in the standard brain and estimating connections between them. The standardization scheme presented here could be combined with other schemes, such as VIB [24], and applied in launching modeling studies of various insect brains.

## 2. Materials and Methods

### 2.1. Histology

Male silkmoths (*Bombyx mori* L., Kinshu and Showa strain hybrids) were used 2–7 d after eclosion. The brains were fixated in 1-2% formaldehyde for 20 h at 4°C. After fixation, they were rinsed in TRIS buffer, dehydrated in an ascending ethanol series with 10 min/step, degreased in methyl salicylate/ethanol to promote antibody penetration for 30 min and rehydrated. After rinsing in TRIS buffer, they were incubated with agitation for 3–7 d at 4°C in TRIS buffer containing 0.5% Triton-X 100 and 1% bovine serum albumin (TRIST-blk) as well as mouse monoclonal anti-*Drosophila melanogaster* synaptotagmin antibody (3H2 2D7 contributed by K. Zinn and obtained from the Developmental Studies Hybridoma Bank developed under the auspices of the NICHD and maintained by the University of Iowa, Department of Biology, Iowa City, IA 52242, USA, at a dilution of 1 : 15–1 : 50 of the concentrate).

After incubation in the primary antibody, the samples were rinsed in TRIST-blk (5 × 15–60 min) and transferred to the secondary antibody (Molecular Probes Alexa Fluor 488 anti-mouse, 1 : 200–1 : 250 in TRIST-blk) for 2 d at 4°C. Finally, samples were rinsed again in TRIST-blk and plain TRIS buffer (5 × 15–60 min), dehydrated, and cleared in methyl salicylate.

Imaging was done in methyl salicylate with a Zeiss LSM510 confocal laser scanning microscope (LSM) and 10×/0.45 or 40×/1.0 oil apochromat objectives. Image data were registered in our database system, BoND [14], for sharing among collaborators.

### 2.2. Method for Constructing a Standard Brain

**Figure 1:**
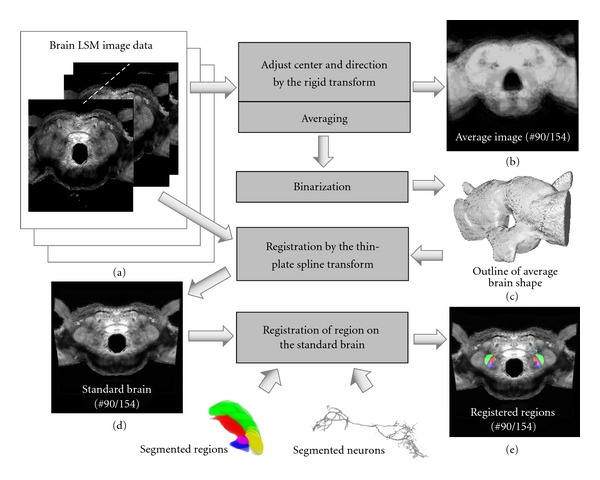
Scheme for constructing the standard brain. (a) Brain LSM image data consisting of multi-page image stacks. (b) Averaged brain image data (section 90 of the set of 154 optical sections of 2 *μ*m thick optical sections is shown). (c) Average shape of the silkmoth brain obtained by binarization and surface modeling. (d) High-resolution brain image was registered by fitting to the standard brain shape using a nonrigid transform. (e) Segmented regions and neurons were registered in the standard brain by a nonrigid transform with defined landmarks.

We developed an original method for constructing a standard brain using confocal LSM brain image data (Figure 1(a)). In our database, there are six whole brain image datasets scanned from both anterior and posterior. Most of these were scanned at low magnification, and are unsuitable for detailed segmentation of brain regions. In our method, we averaged these LSM images aligned by adjusting centers and orientation through translation and rotation to calculate an outline of the average shape (Figure 1(b)). After binarization of the images, the outline of the standard brain was obtained and a polygon model in Wavefront OBJ format was also generated by the image processing software Fiji (Figure 1(c)) [25]. Since we assumed that moth brains are strictly bilaterally symmetrical, 12 brain datasets from six individuals were used to construct the average brain shape.

A high-resolution image dataset of the brain was registered using the thin-plate spline transform of Fiji in the standard brain (Figure 1(d)). The standard brain with internal image was applied for registration of brain regions and neurons. Landmarks, which are reference points in the transform, were assigned to characteristic points with direct correspondence in the two brain image stacks. The thin-plate spline transform based on the landmarks was also used for registration of regions and neurons (Figure 1(e)). In order to apply the transform, it was necessary to assign at least four landmarks. Moreover, it was important that the landmarks were selected evenly in the horizontal and vertical directions to avoid directional biases.

### 2.3. Software for Segmentation and Registration

Several software tools were evaluated and applied in our segmentation and registration scheme. In the first step, neural morphologies were segmented using the ITK-based segmentation software, ITK-SNAP [26] and our own program, KNEWRiTE. ITK-SNAP was most useful as it runs on a number of operating systems (Windows, Linux, and MacOS X) and has many useful functions for extracting objects from multi-layered image data. In particular, automatic segmentation based on the snake algorithm is quite effective for extracting dendritic branching structures.

**Figure 2:**
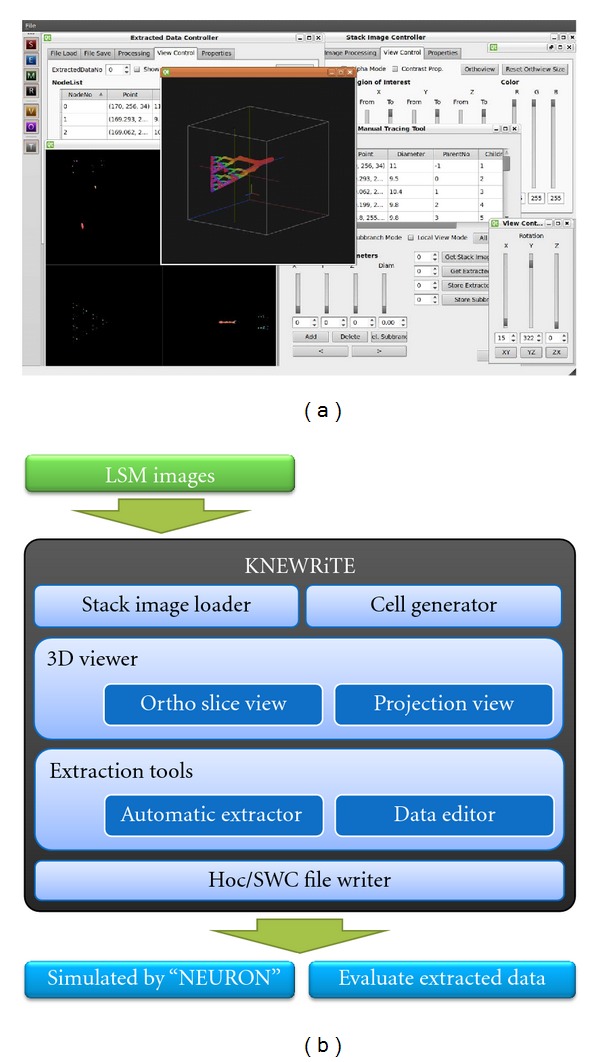
KNEWRiTE, our new software for segmentation of single neuron morphology. (a) Screenshot of the software. The software can trace dendritic and axonal trees automatically, and allows manual editing and addition of segments that are difficult to detect automatically. (b) Structure of KNEWRiTE. The “Stack Image Loader” and “Cell Generator” load LSM image data and separate neurons from background by binarization. The neuron structure is extracted by “Extraction Tools,” an automatic extractor and data editor. The results are exported as image data and a morphological model in SWC format.

KNEWRiTE (http://invbrain.neuroinf.jp/modules/htmldocs/IVBPF/IOSSIM/index.html) was also applied to extract the neuron structure from LSM image data (Figure 2). The software, using Qt (http://qt.nokia.com/) and OpenGL (http://www.opengl.org/) for the GUI, runs on Linux and Windows. It has a function for tracing the branching patterns of 3D dendritic structures based on a region growing approach [27] and also manual tracing. The automatic tracing method is very effective for high-contrast image data without noise, whereas manual segmentation is quite useful for extracting dark and thin objects. A combination of these methods, semiautomatic extraction, was most suitable for our segmentation work.

**Figure 3:**
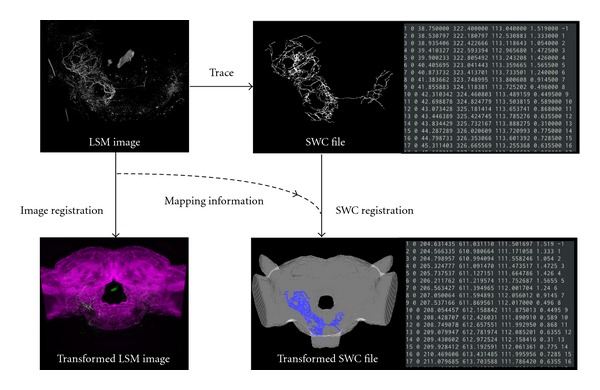
Registration process of SWC data in the standard brain. The neuron morphological model file is obtained by tracing using KNEWRiTE. The LSM image of the brain including the stained neuron is registered by image registration using a nonrigid transform to the standard brain. Our newly developed ImageJ plugin, NeuroRegister, generates a registered SWC file by applying the same transform as for image registration to the data in the SWC file.

Our aim in registering neurons in the standard brain is the construction and approximation of the neuron network in a realistic structure preserving morphological relationships. Neuronal morphologies extracted by KNEWRiTE or ITK-SNAP are stored in SWC file format, which can be used to generate morphological descriptions for various neuronal simulators, such as NEURON [28]. In the registration process of neurons in the standard brain, brain image stacks including neurons can be registered by a thin-plate spline transform in the same way as registering brain regions. However, as no software existed to apply this transform to an SWC file, we developed a new SWC registration plugin module, NeuroRegister (http://invbrain.neuroinf.jp/modules/htmldocs/IVBPF/IOSSIM/index.html) for ImageJ and Fiji. The module was developed based on the “Name Landmarks and Register” plugin module for Fiji. We can apply rigid, affine, and thin-plate spline transforms to objects described in SWC format using almost the same operation as in the original module (Figure 3).

## 3. Results

We obtained the average outline of the moth brain as the basic framework for registration. Then, the neuron morphological models extracted from LSM image data were registered in the standard brain.

### 3.1. Average Outline of the Brain

**Figure 4:**
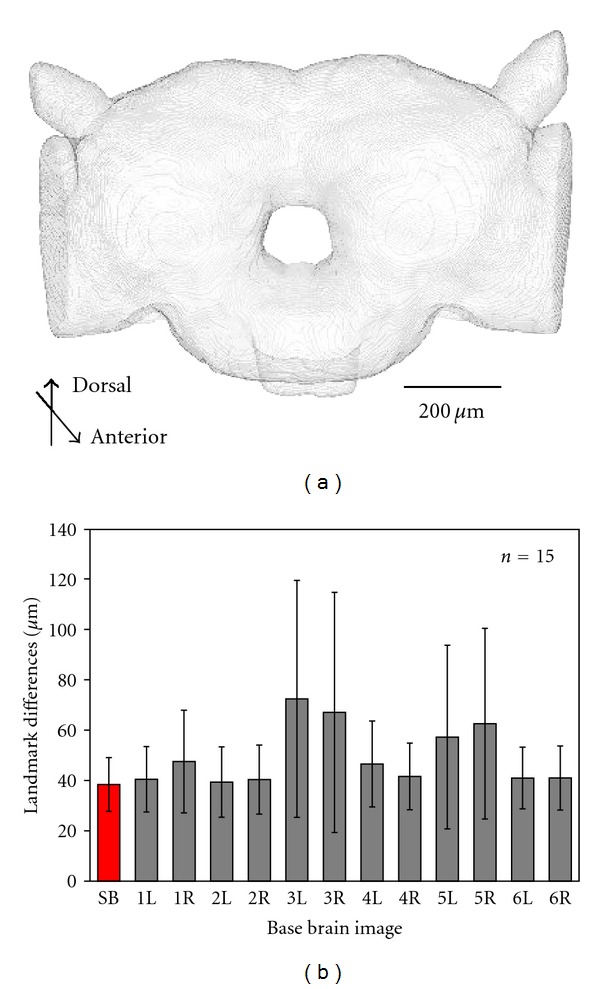
Outline of a base brain and evaluation of variability using the average distance between corresponding landmark points in chosen base brains and all other brain images. (a). Three-dimensional model of the average brain. (b). Euclidean distance is used to measure positional differences of landmarks. The standard brain (SB) was assumed to be bilaterally symmetric, so 15 landmarks in each of the individual samples (*n* = 12 from 6 moths assuming bilateral symmetry, *L* is the left and *R* the right hemisphere) and the standard brain were used for analysis. When assigning the standard brain as the base brain image, the average positional error for landmarks was minimized, resulting in an error of 38.4 ± 10.7 *μ*m. There was no significant difference among groups according to the Tukey multiple comparison test.

The average outline of the brain is very important as a foundation for registering segmented objects in the brain. In this study, the right and left sides of brain images were considered as independent brains by assuming bilateral symmetry. To obtain the standard brain shape, 12 images from six brain samples were used. We defined the center of the esophagus in the slice with the largest brain outline (MS: middle slice) as the origin of the brain coordinate system Five landmark points, namely, the center of the central body (CCB), the centers of the left and right mushroom body calyces (RMC, LMC), and the centers of the left and right antennal lobes, were assigned to apply the transform. A rigid transform was applied to compensate for the difference in position and rotation among the brain image data. Transformed images were averaged and brain regions were extracted as the standard brain shape by separating light and dark areas. The binary image and outline of the standard brain were stored in multi page TIFF format. A polygon based surface model was also generated and stored in Wavefront OBJ format using the “Create Surface” plugin for Fiji (Figure 4(a)).

It is appropriate to use the average shape as the standard brain because morphological data of regions and neurons from different individual brains will be registered in it. To evaluate our standard brain shape, characteristic landmark points from various image slices, which were not used in the rigid transform process, were assigned on the standard brain and each brain image data. In our evaluation, one set of image data including the standard brain was selected as the base brain image. To evaluate the difference in outline shapes, we assigned 15 points, that is, three points on each of the dorsal and ventral outlines in the posterior #50 and middle #80 image slices and two dorsal and one ventral point from posterior image slice #123. These landmarks were selected edges or points clearly seen and identified in every sample. The differences in the coordinates between the base and another image data were measured by Euclidian distance. It was shown that the average distance was minimized with the standard brain image as the base image. Standard deviation was also minimized in this case (Figure 4(b)). It was also shown that based on a Tukey multiple-comparison test, there was no significant difference between the groups. Moreover, the shapes of sample brains were very similar, with the standard brain having the most general size and shape of moth brain of the sample brain images under consideration.

### 3.2. Comparison of Segmentation Methods

**Figure 5:**
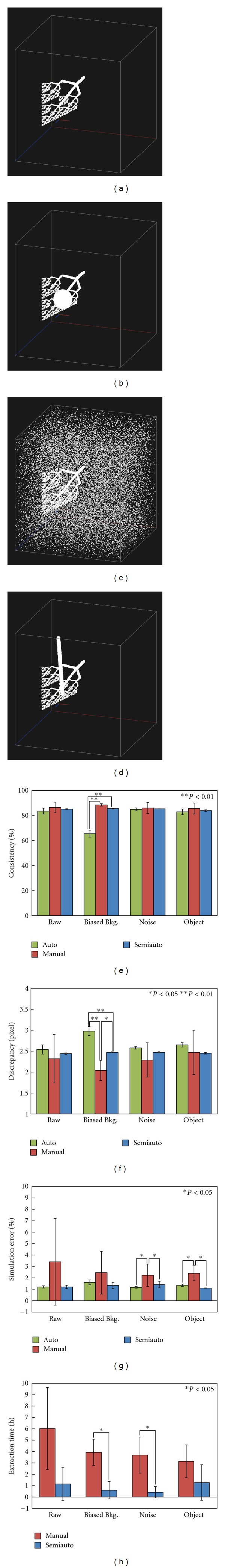
Evaluation of the three extraction methods (auto, semiauto, manual) of KNEWRiTE using four types of artificial 3D neuron images generated by connection of cylinders. (a) Raw: neuron without other objects or noise. (b) Biased background: a large object covers the neuron. (c) Noise: addition of white noise. (d) Object: neuron and overlapping cylindrical object. (e) Mutual consistency: missing existing branches and detecting nonexistent branches. (f) Discrepancy of diameters: discrepancy of diameters between neuron and extracted model. (g) Error of simulation: difference between neuron and extracted model in passive model simulation. (h) Extraction time: time required for extraction. Automatic extraction, which took less than 1 min for each case using conventional PC hardware, is not shown in this graph. The Tukey multiple-comparison test examined differences among groups.

Using the KNEWRiTE software, automatic, manual and semiautomatic methods were selected for efficient and high quality segmentation. To evaluate the performance thereof, we applied these to extract more than three computer-generated arborized objects. Extractions of objects were executed under four different conditions, namely, without other objects or noise denoted by “Raw” (Figure 5(a)), a mixture of large objects denoted by “Biased Background” (Figure 5(b)), with white noise denoted by “Noise” (Figure 5(c)), and with a cylinder object denoted by “Object” (Figure 5(d)).

Results of the extractions were evaluated according to the success rates of segmentation, denoted by “Consistency,” which is the mean of two consistency measurements, one corresponding to missing existing branches and another related to detecting nonexistent branches falsely [29]. It was shown that more than 80% of the structure was extracted correctly using manual and semiautomatic extraction methods (Figure 5(e)). However, the success rate was smaller in the case of automatic extraction for every condition. Moreover, the standard deviation for semiautomatic extraction was fairly small compared with the other methods, which means that the semiautomatic method is not susceptible to the object shape and background noise.

The three extraction methods were also evaluated “Discrepancy,” which is the discrepancy in the diameter between the model and segmentation results (Figure 5(f)). The accuracy of manual extraction was the best, while that of automatic extraction was the worst in most cases. However, the results of manual extraction were strongly dependent on image conditions. In the case of semiautomatic extraction, accuracy was quite high and stable, irrespective of the object shape and image conditions. We also tested our results by applying a passive membrane model to the extracted neuron morphological models. The error in the electrical response was minimized in the case of semiautomatic extraction (Figure 5(g)).

The results show that extraction of neuronal morphology based on automatic extraction with manual adjustment is the best for segmenting neurons for simulation of their dynamical properties. Finally, the elapsed time of extraction was less than 1 min for automatic extraction, but more than 1 h for the semiautomatic method (Figure 5(h)). Nevertheless, it is clear that this is a very efficient way of segmenting neurons compared with the manual method.

### 3.3. Registration of Brain Regions and Neurons

As an attempt to utilize various kinds of brain image data taken for different purposes and individuals in our database, we applied a nonrigid transform to register these in the standard brain. We used a thin-plate spline transform to register high-resolution image data fitted onto the standard brain shape. The standard brain with brain regions can be used for registration of extracted regions and neurons.

**Figure 6:**
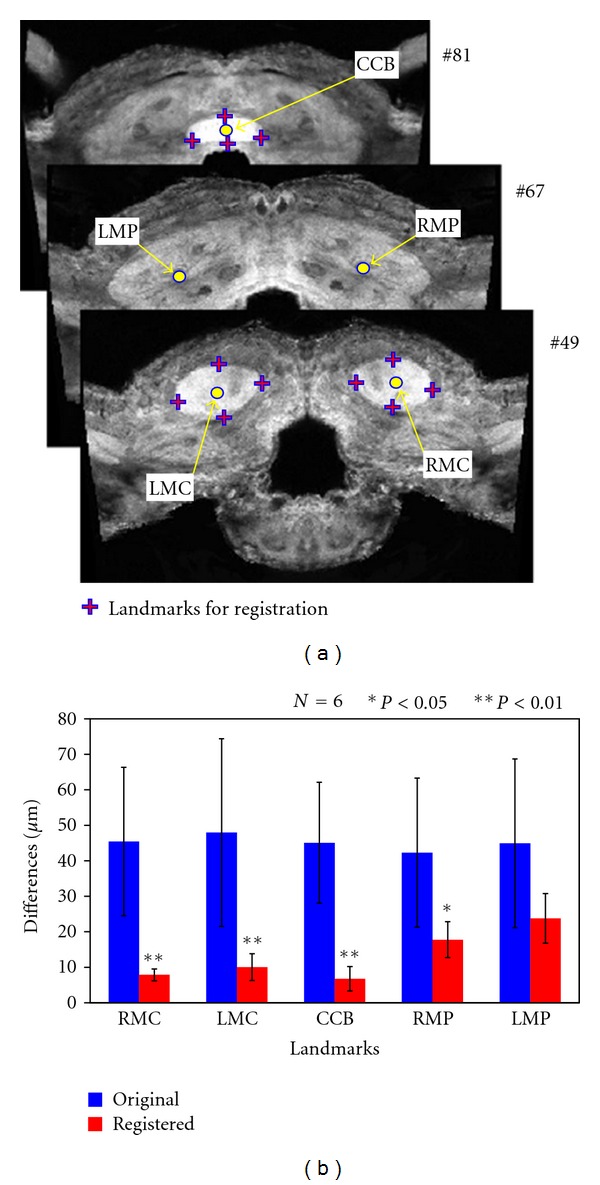
Differences in coordinate values of distinct points before and after registration. (a) Twelve landmarks for registration in the standard brain (cross) were assigned from the edges of three clearly delineated regions, the central body and themushroom body calyces. Six distinct points (circles), the center of the central body (CCB), the centers of the calyces (RMC and LMC), and the peduncles (RMP and LMP) of mushroom bodies were selected to calculate the registration error. (b) Euclidian distances were measured between distinct points in the standard brain and each sample brain before and after registration. The average and standard deviation are plotted for each point. With the exception of LMP, there were significant differences based on the one-way ANOVA before and after registration.

**Figure 7:**
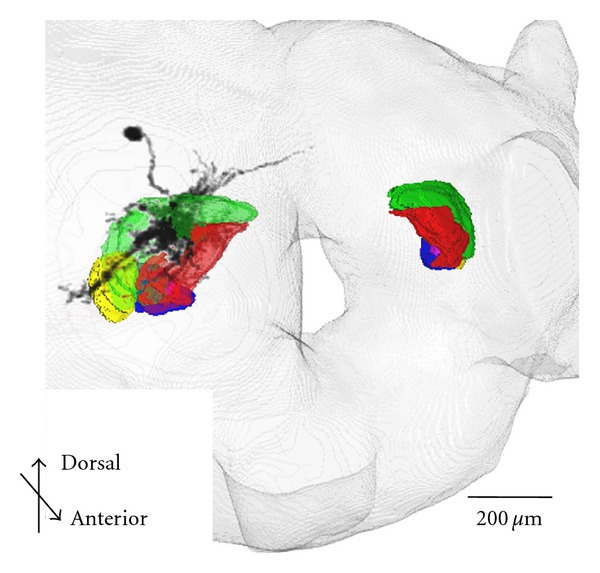
Registered LAL-VPC regions and neurons in the standard brain. LAL-VPC consists of five subregions, labeled in red, green, yellow, blue, and magenta. A neuron (ID 0986 in our database, BoND) arborizing in the LAL-VPC was segmented and registered in the standard brain.

To evaluate the accuracy of our method, we measured the differences in coordinate values of distinct points in the moth brain, namely, the center of the CCB and the centers the calyces (RMC, LMC) and the peduncles of the mushroom bodies (RMP, LMP) between the moth brain were measured between the standard brain and brain images before and after registration. Twelve landmarks were assigned from the edges of clearly segmented regions, such as central body and the mushroom body calyces (Figure 6(a)). The Euclidian distance of distinct points on the standard brain and original brain images was greater than 40 *μ*m, but our registration moved these significantly closer except for LMP by one-way ANOVA. The results show that the average registration errors of the points surrounded by landmarks, RMC, LMC, and CCB, were 7.85, 10.04, and 6.74 *μ*m, respectively (Figure 6(b)). However, the errors of points distant from landmarks, RMP and LMP, remained greater than 15 *μ*m. It is obvious that the accuracy of registration depends on the selection of landmarks, and thus, it is important to assign landmarks close to the regions or objects of interest. In this study, the LAL-VPC regions were manually segmented in the high-resolution image data. By applying the registration process to the segmented regions, these were registered in the standard brain (Figure 7). Segmentation of regions and neurons was a time-consuming process, but computation time for registration using the thin-plate spline transform was less than 10 min for each object using conventional computers. Other regions can be registered in the same way. The average shape and position of regions will be statistically calculated by collecting a larger number of samples.

A great deal of effort was expended in extracting the three-dimensional dendritic structure of neurons from LSM stack images. Our original software, KNEWRiTE, can extract a neural structure from LSM image data by combining automatic and manual processes. We applied this to extract neurons arborized in LAL-VPC regions. Several preprocessing steps were applied to the LSM image data, such as adjusting contrast, using Fiji and ITK-SNAP. After binarizing the image data, neuron morphology was extracted and a morphological model was generated semiautomatically in SWC format using KNEWRiTE. The percentage of extraction using either automatic or manual processes was dependent on various conditions, such as neuron morphology, contrast in neuron images, and other factors related to image quality. In the case of neurons with a simple structure, this was extracted finely without any manual operation. However, more than 10% of the fine dendrites were extracted and connected manually in the case of neurons with thick arborizations. The extracted neuron image data were stored in a TIFF formatted file, and then a polygon model was generated in Wavefront OBJ format.

**Figure 8:**
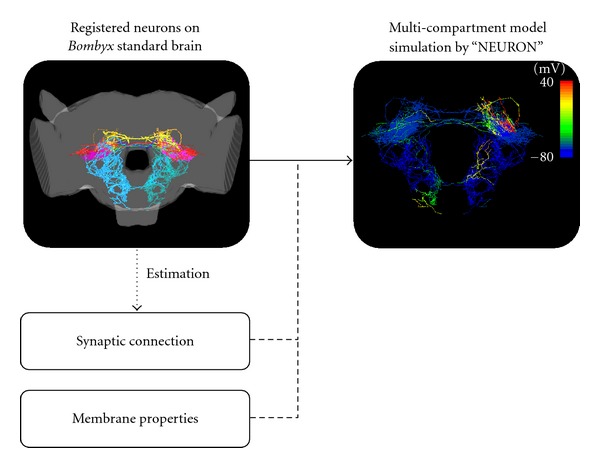
Registration of neurons in the standard brain and applications thereof. It is possible to estimate the strength of synaptic connections by the volume of overlap of two neuronal branches registered in the standard brain. Network model simulation of the silkmoth brain with neuronal morphologies based on experimental data, electrical properties, and synaptic connections are implemented and executed on the supercomputer.

Segmented neurons were registered in the standard brain by the thin-plate spline transform of Fiji. To apply this registration, more than four of the landmark points had to be assigned on the brain image involving segmented neurons and on the standard brain. Our original plugin software, NeuroRegister, was applied to transform and register the neuron morphologies in SWC format. It was confirmed that registration was adequate for analyzing the projection area and the overlap of neuronal projections of different neurons (Figure 8).

## 4. Discussion and Conclusions

Standard morphological atlases have come a long way since compilations of serial sections for reference and identification of brain areas. In the form of a standard brain atlas, they permit the accumulation of experimental data while preserving morphological relationships and simplify comparative analyses between species. Besides its use as a database-like tool, we aim to use our silkmoth standard brain as a platform for large-scale neural network simulations.

When starting to construct a standard brain, care must be taken to calculate the average outline shape as precisely as possible. In our scheme, we assigned the center of the esophageal foramen in the middle slice in the anteroposterior direction as the coordinate origin of the brain, since the edge of the esophageal foramen was clearly seen and its center was easily obtained. Then, we adjusted each brain image stack using translation and rotation. The size and shape were unchanged in this process. The average image stack of the brain was calculated by averaging the grey scale value in each pixel for all 12 LSM brain image stacks. This protocol is presented to construct an average brain outline that can serve as a measure for the differences in shape and size among individual brains. We evaluated the shape of the standard brain by calculating the distance of corresponding landmarks for all individual samples. The average landmark distance between single samples and the standard brain was less than 40 *μ*m, which corresponds to the difference in shape among individuals (Figure 5).

Any brain image data set can be registered by setting landmarks on the outline of the brain. We have various high-resolution LSM images of the brain, and these images are registered in each slice of the standard brain in a way comparable to texture mapping. Extracted objects such as neurons, neuropils, and tracts can be registered by applying a transform to the standard brain from sample brain image data containing these labels. Through this approach, we can obtain and integrate the registration results of neuropils. A further goal is to implement semiautomatic or automatic segmentation and registration procedures for neuropils, possibly applying various transform and deform techniques already in use in medical image processing.

In our scheme, morphological models of neurons are reconstructed from confocal image data of neurons. Extracted neuron images are registered into the standard brain by applying a nonrigid transform. Morphological neuron models in SWC format are also registered using our ImageJ plugin module (Figure 6). Morphological properties of neurons are modeled and registered by our proposed scheme, and then geometrical properties of groups of neurons, such as the overlap of axonal and dendritic trees, provide estimated information concerning the position and strength of synaptic connections. Further information, such as the types of ion channels, their dynamics and distributions along neurites, will be very helpful for model simulation of neuronal properties.

We are currently implementing our standard brain protocol and software environment using a supercomputer. We are constructing a platform to integrate morphological and physiological properties measured in a large number of individual experiments. High-performance computing for neuronal modeling and simulations in conjunction with experiments based on the standard brain could become very powerful tools for a new era of integrative computational neuroscience research.
